# Comparative Effect of two Types of Surface Treatments on Shear Bond Strength of New Composite to Old Composite

**DOI:** 10.30476/DENTJODS.2021.84910.1106

**Published:** 2021-12

**Authors:** Seyedeh Maryam Tavangar, Reza Tayefeh Davalloo, Tayebeh Rostamzadeh, Farideh Darabi, Seyed Mohammad Ali Mirabolghasemi, Reza Ahmadi

**Affiliations:** 1 Dept. of Operative Dentistry, Dental Sciences Research Center, School of Dentistry, Guilan University of Medical Sciences, Rasht, Iran; 2 Dept. of Operative Dentistry, School of Dentistry, Guilan University of Medical Sciences, Rasht, Iran; 3 General Dentist, Rasht, Iran

**Keywords:** Poly methyl Methacrylate, Composite Resins, PermaSeal, Chloroform, Dental Bonding

## Abstract

**Statement of the Problem:**

Composite restoration failures may occur because of different factors. In these situations, the repair of a composite restoration has many advantages over replacement such as saving time, lower cost, and lower risk of excessive removal of sound tooth structure and subsequent pulp exposure.

**Purpose:**

The purpose of this *in vitro* study was to evaluate the effects of two surface treatments on shear bond strength (SBS) of new composite to old composite.

**Materials and Method:**

In this *in vitro* study, 60 composite discs were fabricated using a plexiglass mold measuring 4 mm in thickness and 7 mm in diameter, and were randomly divided into three groups (n=20). In group 1, the bonding procedure was done with no modification. After roughening of one surface in all remaining samples, chloroform (CHCl3) was applied on the surface of samples in group 2 and phosphoric acid 35% was applied on the surface of the samples in group 3. PermaSeal was then applied in all samples and new composites were bonded to the surface. The samples were stored in distilled water for one week and were then subjected to 500 thermal cycles and shear bond strength between two layers of composite and mode of failures were evaluated.

**Results:**

The lowest and the highest SBS values of repair composite to old composite were noted in groups 3 and 1, respectively and this difference was statistically significant (*p*< 0.05).The difference between groups 1 and 2 was not significantly different (*p*= 0.197). The mode of failure was mixed in all samples of groups 2 and 3 and cohesive in group 1.

**Conclusion:**

After grinding, the surface treatment with phosphoric acid did not increase the SBS of new composite to old composite, while chloroform increased the SBS almost to the level of the baseline in control group.

## Introduction

Despite the modifications made in the formulation of composite resins, their high technical sensitivity leads to many failures in the clinical setting, especially in the posterior teeth [ 1 ]. Repair of a composite restoration with chipping, wear, or discoloration may serve as a low-cost, durable alternative to restoration replacement [ 1 ]. Some repairs can be performed without the need to use local anesthesia, and may be less distressing for the patient compared with the instances that replacing the filling is necessary [ 2 ]. Replacement of composite restorations with small defects can be time-consuming and has a high risk of excessive removal of the sound tooth structure and subsequent pulp damage. Thus, the repair of defective restorations instead of their replacement can be considered as a favorable [ 3 ] and more conservative [ 4 ] approach. Repair of composite restorations is often accomplished by placing new composite over the old composite by macro- and micromechanical retention. Macromechanical retention can be created by the preparation of undercuts in the old restoration, which can also improve the resistance form [ 5 ]. Micromechanical retention can be obtained by preparation with a coarse diamond bur [ 5 ] and phosphoric acid etching [ 6 - 7 ] and air abrasion with aluminum oxide particles with/without using silane coupling agent and resin bonding systems [ 8 ].

Considering the repair of composite restorations, some studies have reported many problems; the polishing procedure reduces the reactive groups and makes in-organic filler particles exposed to the surface which may subsequently reduce the bonding ability and prevent achieving a durable and strong bond between the old polymerized composite and the new composite resin [ 8 ]. Consequently, the repair bond strength may become lower than the primary bond strength by 25% to 80% [ 1 ].

Bonestine *et al*. [ 9 ] employed various repair preparation methods including no treatment (control group), phosphoric acid, diamond bur, air abrasion, silane primer combined with a diamond bur treatment and showed that the highest shear bond strength (SBS) belonged to the diamond bur group. They included that the lowest SBS was related to the phosphoric acid method, which was not significantly different from the control group [ 9 ]. Another study investigated the effect of different surface treatment methods, including no treatment, air abrasion with 50-μm aluminum oxide particles, irradiation with Er:YAG laser beams, roughening with coarse-grit diamond bur+35% phosphoric acid and etching with 9% hydrofluoric acid for 120 seconds on the SBS in composite repair [ 10 ]. The study showed that SBS of controls was significantly lower than the other groups and the differences between the other groups were not significant [ 10 ].

The study of Hemadri *et al*. [ 11 ] also found no difference in the SBS among various surface treatment methods including no surface treatment, abraded with diamond bur, air abraded (sandblasted) with 50 µ aluminum oxide particles. Unfortunately, a standard and exclusive method for creating a durable and long-lasting bond between the old polymerized composite and the new restorative resin has not yet been reported [ 12 ]. The same problem exists in the repair of fractured denture bases and worn artificial teeth with composite resins. Evidence shows that successful denture repair (bonding of the two fractured pieces with a repair material) depends on the adhesion phenomenon, and treatment of bonding surfaces is highly essential to guarantee a long-term clinical service [ 12 ]. Proper surface treatment ensures high repair bond strength and decreases stress accumulation [ 12 ]. Considering the successful results of studies about application of chloroform in repair of denture base [ 12 - 13 ] and the presence of bisphenol A-glycidyl methacrylate (bis-GMA) in the formulation of composite resin and lack of sufficient study in the field of repair of composite restoration with this material, the purpose of this study was to assess the shear bond strength of old composite to new composite when using chloroform and phosphoric acid 35% as surface treatment.

## Materials and Method

This *in vitro* study, evaluated 60 composite discs fabricated in a plexiglass mold measuring 4 mm in thickness and 7mm in diameter. The mold was first filled with A1 shade of Amelogen (Ultradent products Inc; USA) composite in two increments of 2mm (Table 1). Each layer was separately light-cured for 20 seconds using a LED light-curing unit (Bluedent LED Smart; Bulgaria). Final curing was performed for another 40 seconds by continuous irradiation of light with an intensity of 1300 mW/cm2. The light intensity was measured by a radiometer (LM100; Digi Rate) before the study and after preparation of each group. Then the fabricated samples (n=60) were randomly divided into three groups (n=20). 

**Table 1:** Specifications of consumed materials

	General specifications	Manufacturing factory	Used material
1	Light-cure composite with Bis-GMA base filler of 76% by weight and 61% by volume. The average filler particle size of 0.7 microns	ULTRADENT, Products.inc, USA	Amelogen Plus, Composite restorative material
2	Non-filler resin with methacrylate base	ULTRADENT, Products. inc, USA	PermaSeal, Composite sealer
3	Phosphoric acid 35%	ULTRADENT, Products.inc, USA	Ultra-Etch
4	CHCL3 100%	KIMIA.CO, IRAN	Chloroform

**Figure 1:**
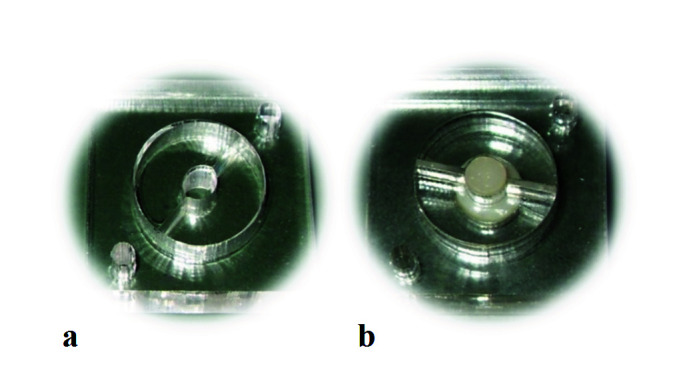
**a:** Plexiglass mold, **b:** plexiglass mold after composite placement

In group 1, the composite did not receive any surface treatment and immediately the second mold was placed over the first mold via two metal rods and three layers of the new composite. The first layer was 1mm and then two increments of 1.5mm were immediately applied on its surface using another plexi glass mold with 4mm thickness and 4mm diameter (Figure 1). 

Each layer was separately light-cured for 20 seconds. After removing the samples from the molds, they were cured again for 40 seconds (as positive control group). Then, all samples were placed in distilled water at room temperature for one week (groups 1, 2, 3). After wards one surface of each remaining sample (n=40) in group 2 and 3 was roughened by a flame diamond bur (Teezkavan, Tehran, Iran).All samples were then placed back in the original mold and were randomly divided into two groups (n=20). 

In group 2 (n=20), chloroform (CHCl3; Kimia, Iran) was used for surface treatment of samples using a microbrush (TPC, PRC) for 5 seconds and was then rinsed with water for 15 seconds(as recommended by manufacturing company) and dried with air spray [ 12 ].

In group 3, phosphoric acid 35% (Ultradent Products Inc., USA) was applied on the surface of samples with a microbrush for 20 seconds, then rinsed for 15 seconds and air-dried.

Then according to the manufacturer's instructions, PermaSeal (Ultradent Product Inc., USA) was applied on the surface of samples of group 2, 3. This material was rubbed on the composite surface for 5 seconds, thinned with air spray, and cured for 20 seconds. A plexiglass mold (with 4mm diameter and thickness) was fixed as explained earlier, and a new layer of composite was added into the mold (as in group 1). The samples were stored in distilled water for one week, and thermocycling was performed in 500 thermal cycles in all samples of three groups (5-55°C), 30 seconds dwell time and a transfer time of 10 seconds. Then the custom-made jigs were mounted to a Universal Testing Machine (STM20; Santam, Tehran, Iran). A test was run at a crosshead speed of 0.5 mm/min until failure. To express the bond strength in megaPascal (MPa), the load upon failure was recorded in Newtons (N) divided by bond area (mm^2^) [ 14 ]. 

The presence of fractured samples was observed under a stereomicroscope (TR30 SZXZ, Olympus) with magnification (25×) to analyze the mode of failure. 

### Statistical analysis

The Kolmogorov-Smirnov test was applied to assess the normal distribution of data. One-way ANOVA was used to compare the mean SBS of the groups, while pairwise comparisons were carried out using Tukey's LSD test.

## Results

**Figure 2:**
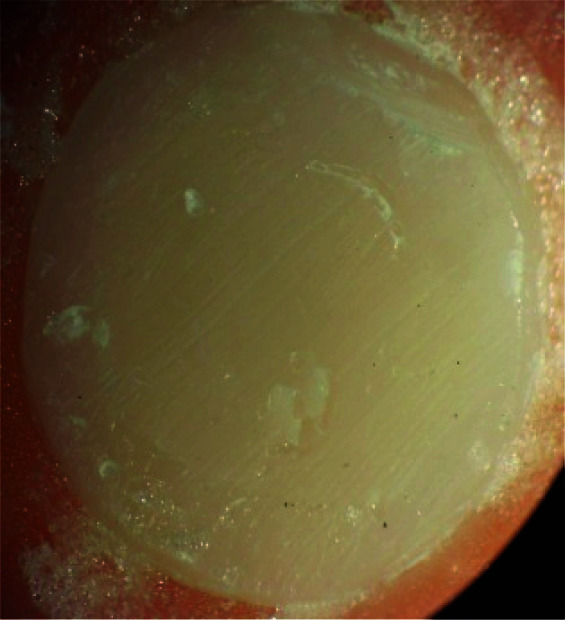
Mixed failure mode

Table 2 shows the mean SBS of three groups. The results (Table 2) showed that the SBS of the new to old composite was minimum in group 3 and maximum in group 1 (control) (Figure2). The SBS of the three groups was significantly different (*p*< 0.05, Table 3). 

**Table 2:** Description of the mean and standard deviation values (SD) of the bond strength

Group	Surface treatment material	Number	Mean (Mega Pascal)	Standard deviation	95% confidence interval		Minimum	Maximum
					Upper limit	Lower limit		
1	Without any surface treatment	20	17.75	3.14	19.18	16.32	11.05	22.52
2	Bur+Chloroform+Bonding+ Composite	20	16.28	3.69	17.96	14.60	6.91	21.26
3	Bur+Phosphoric Acid+Bonding+ Composite	20	13.29	4.11	15.22	11.37	7.36	20.10

**Table 3:** Pair wise comparison of surface treatment materials used in terms of the bond strength

	Differences of means	The standard error	*p* Value	95% confidence interval for difference of means	
				Low limit	Upper limit
Compare Group 1 with 2	1.47286	1.12940	0.197	-0.7871	3.7328
Compare Group 1 with 3	4.45840	1.14343	0.000	2.1704	6.7464
Compare Group 2 with 3	2.98555	0.14343	0.011	0.6976	5.2735

LSD stands for the least significant difference

**Figure 3:**
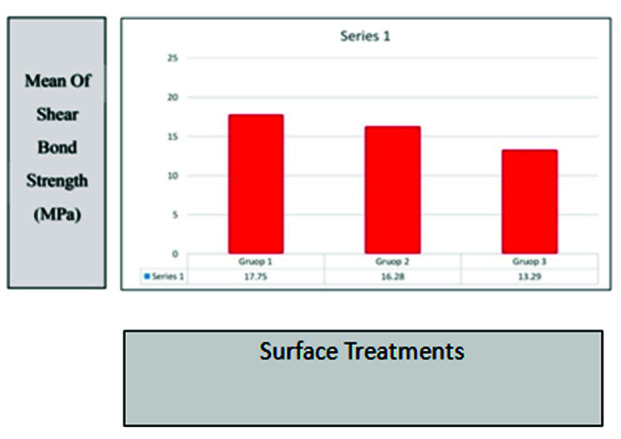
Mean of shear bond strength

Thus, pairwise comparisons were carried out using post hoc LSD test, which showed that the mean SBS of group 3 was significantly lower than that of groups 1 and 2 (*p*< 0.05) while the mean SBS of groups 1 and 2 was not significantly different (*p*= 0.197). The mode of failure was mixed in all samples in groups 2 and 3 (Figure 3) while it was cohesive in group 1, which showed that the mode of failure of the control group was different from that of groups that received surface treatments.

## Discussion

Composite resins are commonly used restorative materials that well preserved the tooth structure, are durable and provide optimal esthetics [ 5 ]. Replacement of a defective restoration is time-consuming and associated with the risk of excessive removal of the sound tooth structure and subsequent pulp damage [ 15 - 16 ]. The problem often encountered in the repair of composite restorations is that the active methacrylate groups on the composite surface that are responsible for bonding of composite layers to each other often decrease after polymerization, finishing and polishing and long-term clinical service in the oral environment [ 17 - 21 ]. Evidence shows that the bond strength of new composite to old composite may be lower than the baseline bond strength by 25% to 80% [ 6 ]. Several techniques are available to create a strong bond between the new and old composite using different surface treatments, such as the creation of mechanical interlocking by use of diamond burs and sandblasting, etching by phosphoric acid or hydrofluoric acid and chemical bonding by use of silane and adhesive [ 22 ]. However, no consensus has been reached on a standard method for this purpose. 

The intraoral surface pretreatment of an old resin composite has two purposes: (1) to remove the superficial layer altered by saliva to expose a clean, higher energy composite surface and (2) to increase the surface area through creation of surface irregularities [ 23 ].

Etching is a routine step in resin composite repair procedures for removal of debris from surface after grinding [ 24 ]. Moreover, elimination of surface debris and filler exposure enhances the surface energy and wettability of the surface [ 25 ].

Problems associated with repair bond strength also exist in the repair of fractured denture bases or worn artificial teeth with composite resin. Shen *et al*. [ 12 ] suggested surface treatment of denture base with chloroform for 5 seconds to obtain higher bond strength. Chloroform is a strong solvent for polymethyl methacrylate. They showed that the application of chloroform for 5 seconds removed debris from the surface of old acrylic resin, created a rough surface, and enhanced the bond strength of new acrylic to the old acrylic base [ 12 ]. 

A previous study showed that the application of chloroform for 5 seconds in repair of denture base results in the dissolution of debris on the surface of aged acrylic resin and creates a rough surface that increases the repair bond strength of new acrylic resin to aged acrylic resin [ 12 ]. On the other hand, a previous study on repair bond strength of composite resin to artificial acrylic teeth of a removable partial denture revealed that surface treatment with chloroform created more porosities on denture teeth and enabled better engagement and interlocking of filler-free bis-GMA resin with denture teeth [ 13 ]. Scanning electron microscopic assessment of the surface of acrylic resin following the application of chloroform shows that following the immersion of acrylic resin in chloroform for 120 seconds, small porosities form on the surface [ 12 ]. Chloroform is the most commonly used solvent in the endodontic retreatment of teeth to eliminate the root filling materials (gutta-percha and sealer) in the clinical setting [ 26 - 30 ]. According to the American Food and Drug Administration, the use of chloroform is banned in medications and cosmetic products [ 16 , 28 , 31 ] since its frequent direct contact with skin is considered carcinogenic [ 31 - 32 ]. However, its use in dentistry has no legal limitation, and carcinogenicity of its dental applications has not been confirmed [ 32 ].

Given that resin composites have also polymethyl methacrylate in their composition (like denture base material), the effectiveness of chloroform on surface treatment of old composite restorations and improvement of repair bond strength can be explained in this way.

The results of the current study indicated that surface treatment of the composite resin with chloroform (group 2) compared to the conventional method (phosphoricacid; group 3) significantly increased the SBS of new to old composite. Application of chloroform increases the surface roughness and enables better penetration of unfilled resin (PermaSeal) into the porosities of the old composite, thus yielding higher bond strength. 

The SBS value in the group 1 was higher than that of group 2 but not significantly; this finding indicates that surface treatment with chloroform significantly increases the SBS of new to old composite. The SBS of group 3 was significantly lower than that of groups 1 and 2, which was in agreement with the results of Lucena-Martín C *et al*. [ 1 ] and Gupta *et al*. [ 22 ]. Bonstein *et al*. [ 9 ] reported that surface treatment with phosphoric acid could not improve repair bond strength values.

This finding can be attributed to the cleaning effect of acid-etching (phosphoric acid) of the surface of the old composite [ 6 ], increased surface free energy [ 7 ], and inability to create micromechanical retention. 

In the present study, the samples were inspected to determine the mode of failure. The study results showed that the mode of failure was cohesive in group 1 and mixed in groups 2 and 3. This result implies that cohesive bond strength was higher than the adhesive bond strength. Cohesive failure in group 1 was expected considering the presence of unsaturated double bonds on the surface of the old composite and optimal chemical bonding of the new composite to the old composite. 

## Conclusion

Surface treatment of the old composite resin with grinding and phosphoric acid did not increase the SBS of the new composite to old composite but surface treatment with chloroform can increase this bond strength.

## Conflict of Interest

None declared.
